# Impact of a Decision-Support Interface on Mental Workload and Stress in Prehospital Stroke Triage: A Randomized Crossover Trial

**DOI:** 10.5811/westjem.61934

**Published:** 2026-09-05

**Authors:** Yu-Chi Lu, Sung-Chun Tang, Li-Kai Tsai, Jiann-Shing Jeng, Yu-Ching Lee, Ming-Ju Hsieh

**Affiliations:** * National Tsing Hua University, Department of Industrial Engineering and Engineering Management, Hsinchu, Taiwan; † National Taiwan University Hospital, Stroke Center and Department of Neurology, Taipei, Taiwan; ‡ National Taiwan University Hospital, Department of Emergency Medicine, Taipei, Taiwan

## Abstract

**Introduction:**

Prehospital triage challenges often lead to suboptimal choices and delayed treatment for stroke patients. The objective of the study was to evaluate whether a decision-support interface to assist in stroke triage to an appropriate hospital could reduce emergency medical technicians’ (EMT) mental workload.

**Methods:**

This randomized crossover study was conducted between February and March 2025. Each participant completed two 12-minute simulated trials in randomized order: current dispatch orders without decision support and a decision-support interface. We assessed mental workload—the mental effort required to make hospital transport decisions under time pressure during prehospital stroke triage—using heart rate variability (HRV) metrics and the National Aeronautics and Space Administration Task Load Index (NASA-TLX). System usability was assessed using the System Usability Scale. The primary outcome was the root mean square of successive differences for each participant after completing the two trials. The secondary outcomes included the differences of NASA-TLX, System Usability Scale, and other heart rate variability parameters, including mean R-R interval (meanRR), mean heart rate, percentage of successive R-R intervals that differ by more than 50 milliseconds, low-frequency power (LF), high-frequency (HF) power, and the LF/HF ratio. Subgroup analyses examined differences by sex, education, age, and years of service.

**Results:**

A total of 35 emergency medical technician (EMT)-intermediate participants (mean age, 27.5 [5.3] years) completed the study. For the primary outcome, the root mean square of successive differences increased significantly (28.1 [13.0] vs 32.4 [13.6] milliseconds; mean difference, 4.3 milliseconds [95% CI, 1.57–6.99]; *P* < .01), indicating reduced mental workload when the participants used the decision-support interface. For the secondary outcomes, mean heart rate (77.5 [8.6] vs 73.8 [10.6], *P* < .01) decreased, mean R (784.2 [89.5] vs 808.9 [87.3], *P* < .01) increased, and HF power (370.1 [291.7] versus 482.6 [433.9], *P* = .02) increased, suggesting reduced mental workload when the participants used the decision-support interface. The mental demand scores in NASA-TLX decreased from 62.5 (14.6) to 35.5 (17.0). Emergency medical technicians with 1.0–1.5 years of service reported higher System Usability Scale scores than those with 1.5–3.0 years (84.4 versus 71.3, P = .02), and 82.9% of participants rated the system as acceptable (System Usability Scale score ≥ 70).

**Conclusion:**

This study demonstrated that integrating a web-based decision-support interface effectively reduces mental workload and psychological stress among EMTs in stroke triage.

## INTRODUCTION

According to the World Health Organization, stroke was the second leading cause of death globally in 2019, accounting for 11% of all deaths, following ischemic heart disease at 16%.^1^ Given its substantial global burden, optimizing timely access to effective stroke interventions remains a critical public health priority. Ischemic strokes constitute approximately 87% of all stroke cases, highlighting their substantial clinical importance.^2^ Much research focuses on acute ischemic stroke (AIS) patients, including scenarios both with and without large vessel occlusion. After intravenous thrombolysis was found to improve outcomes in patients with ischemic stroke, endovascular thrombectomy was also proven to be beneficial for patients with large vessel occlusion.^3^ Patients with AIS have better outcomes if the time is reduced between onset and receiving treatment to reperfuse the brain tissues.^4^,^5^

To minimize the time window from stroke onset to treatment for AIS patients, the decision-making process for hospital transport must also consider additional factors such as transport time, door-to-treatment duration, and necessary diagnostic testing.^6^ However, not all hospitals were equipped to provide endovascular thrombectomy, and interhospital transfer caused significant delays between the time patients were last seen normal and the initiation of endovascular thrombectomy, which was associated with poor outcomes.^7^ Therefore, it is recommended that prehospital personnel use screening tools to identify patients with large vessel occlusion and transport them directly to hospitals capable of performing endovascular thrombectomy.^8^

In our city, emergency medical technicians (EMT) employ the Cincinnati Prehospital Stroke Scale and the Gaze-Face-Arm-Speech-Time (G-FAST) test to identify patients suspected of having a stroke, including those with a potential large vessel occlusion.^9^ While dispatch systems offer predefined transport routes before EMTs arrive at the scene, they do not consider real-time patient conditions or treatment eligibility, potentially resulting in suboptimal hospital selection, resource concentration at tertiary centers, and treatment delays. To address this challenge, decision-support interfaces may support more accurate triage decisions by integrating real-time clinical and transport information into clear, actionable guidance for EMTs.

Although interface design and decision-support tools have been widely applied in other domains, few studies have investigated their role in reducing the specific decision-making burden on EMTs during stroke triage, which represents a significant gap in current research on prehospital care. This study aims to evaluate a hospital selection interface developed from historical stroke registry data, designed to support EMTs in selecting the most appropriate hospital for suspected stroke patients.^10^ Unlike studies focusing solely on interface usability, our objective was to assess whether an integrated system—comprising a predictive model, user interface, and field validation—could reduce the mental demands associated with hospital selection. By linking EMTs’ mental workload reduction to emergent stroke transport system, our goal was to provide empirical evidence on improving real-world decision-making efficiency and resource distribution in stroke emergency care.

**Population Health Research Capsule**

What do we already know about this issue?

*Prehospital stroke triage is complex and may increase emergency medical technicians’ (EMT) mental workload, potentially leading to treatment delays*.

What was the research question?

*We sought to determine whether a decision-support interface reduces EMTs’ mental workload during stroke triage*.

What was the major finding of the study?

*The root mean square of successive differences increased by 4.3 milliseconds (95% CI, 1.57–6.99; P < .01) with decision support, indicating lower workload*.

How does this improve population health?

*Decision-support tools that reduce EMTs’ mental workload may improve stroke triage and facilitate timely access to appropriate care*.

## METHODS

### Study Design

This randomized crossover study adopted a within-subject design with two experimental conditions to evaluate the impact of a digital decision-support interface on prehospital stroke transport decision-making. The study was conducted and reported in accordance with the Consolidated Standards of Reporting Trials (CONSORT) guidelines.^11^ To mitigate potential order effects, participants were randomly assigned to one of two trial sequences using a Latin square design. Each participant completed both trial conditions—Trial A (current dispatch orders) and Trial B (the proposed decision-support interface). Objective physiological indicators and subjective workload assessments were collected to evaluate participants’ mental workload and user experience.

In both trials, participants were presented with a decision-making test sheet based on standard operating procedures followed by EMTs when managing suspected stroke cases. Each test involved eight simulated patient scenarios, with participants assessing one patient at a time in sequence. They proceeded to the next scenario only after completing a decision for the current case, continuing this process until the 12-minute time limit was reached. For each case, decisions were made based on the provided G-FAST score and relevant patient data. To minimize potential learning effects, each scenario varied in terms of location, symptom-onset time, and G-FAST results. The simulation environment and presentation format were held constant across both trials; only patient-specific variables differed. A video simulation was shown once before the first trial to establish contextual understanding.

### Study Setting and Participants

This study was conducted between February and March 2025. The experimental setting replicated real-world prehospital stroke triage scenarios using standardized patient cases, verbal instructions, and audio distractions to simulate decision-making under pressure. All participants were certified EMTs with prior training in stroke management. The emergency medical service (EMS) system from which participants were recruited is operated by the city’s fire department and comprises a mixed one-tier and two-tier system, including 41 basic life support units and four advanced life support units. There are approximately 1,200 EMTs in the city. 12 During the study period, approximately 12% were EMT-paramedics, while the remainder were EMT-intermediates. The EMT-intermediates receive 280 hours of training, whereas EMT-paramedics undergo 1,280 hours of training. Most patients with suspected stroke were managed by EMT-intermediates.^13^

According to internal evaluations conducted by the fire department, EMTs with less than three years of field experience tend to have higher error rates, with decision-making performance improving markedly after the third year. Based on this empirical observation, we recruited EMTs with fewer than three years of service to focus on early-career personnel. Participants were recruited via announcements and screened for eligibility, including no history of arrhythmia or cardiac-related medication use. All procedures were approved by the research ethics committee of National Tsing Hua University (IRB No. 11310HE165), and written informed consent was obtained from all participants.

### System Overview and Interface Design

The decision-support system evaluated in this study was developed based on a previously proposed risk-averse optimization model for prehospital stroke triage.^10^ The model estimates outcome probabilities for potential hospital destinations by accounting for transport time, treatment capabilities (intravenous thrombolysis/endovascular thrombectomy), and individual patient characteristics. Building on this model, we implemented a user-oriented interface designed to support EMTs in time-sensitive decision-making tasks.

To support novice EMTs who often overlook critical steps under pressure, the interface was designed as a single-page, stepwise workflow. Users are guided through a structured, checklist-style input process that includes G-FAST items, symptom-onset time, and location. Based on the predicted probability of the patient receiving appropriate treatment, the system then dynamically ranks hospital options. The results are displayed with geolocation and visualized treatment time distributions, incorporating eligibility for thrombolytic therapy, transport duration, and hospital capabilities.

Unlike current dispatch forms, the interface integrates clinical decision factors and presents them with visual cues and outcome probabilities. This presentation strategy was intended both to enhance usability and to increase trust in the system’s recommendations by making its logic transparent.

### Outcome Measures

In this study, mental workload was defined as the mental effort required to process clinical information and make hospital transport decisions under time pressure during prehospital stroke triage. We evaluated mental workload during prehospital stroke decision-making, assessed objectively through heart rate variability (HRV) and subjectively via the National Aeronautics and Space Administration Task Load Index (NASA-TLX). The HRV parameters, established indicators of autonomic regulation and mental workload,^14^,^15^ included root mean square of successive differences, mean R-R interval (mean RR), mean heart rate (mHR), percentage of successive R-R intervals that differ by more than 50 milliseconds (pNN50), low-frequency power (LF), high-frequency power (HF), and LF/HF ratio. We used root mean square of successive differences as the primary outcome and other HRV parameters as secondary outcomes. The NASA-TLX evaluated six workload dimensions: mental demand; physical demand; temporal demand; performance; effort; and frustration.^16^ In addition to NASA-TLX, the secondary outcomes included system usability, measured using the System Usability Scale, a validated instrument widely applied to assess perceived usability of digital interfaces.^17^

### Measurements Tools

Physiological signals were recorded using the Polar H10 chest-strap sensor (Polar Electro Oy, Kempele, Finland), a validated device transmitting mean RR via Bluetooth. The RR interval is the time between two consecutive R-waves on an electrocardiogram (ECG). This device has demonstrated high accuracy and consistency compared to ECG in both resting and exercise conditions.^18^ The RR intervals were recorded continuously throughout each trial, and HRV metrics were calculated from a 5-minute segment beginning with the second patient scenario using standard time-domain and frequency-domain algorithms.^19^

Subjective workload was measured using the NASA-TLX, a validated multidimensional tool that includes six dimensions rated from 0 to 100 in this study. A pairwise comparison matrix determined the weighting of each dimension, generating a weighted overall workload score after each trial.^16^

System usability was assessed post-experiment using the System Usability Scale questionnaire. Participants rated their experience with the decision-support interface on a five-point Likert scale, resulting in scores ranging from 0 to 100, with higher scores indicating superior usability. According to the scale’s interpretive guidelines, scores below approximately 50 are considered not acceptable and correspond to an “F” grade, often described as *poor* or *worst imaginable* usability. Scores between 50 and 70 fall within a marginal acceptability range (grades D–C), typically interpreted as *“okay”* usability. Scores above 70 are generally regarded as acceptable, with values between 70 and 80 corresponding to “good” usability (grade C–B). Scores exceeding 80 indicate excellent usability, and those above 90 are associated with “best imaginable” systems (grade A), reflecting outstanding user experience and strong usability performance.^20^ The questionnaire was administered once, upon completion of both trials, with participants instructed to evaluate only their experience using the interface, independent of trial order.

### Sample Size Estimation

We hypothesized that the root mean square of successive differences would be significantly higher in Trial B (the proposed decision-support interface) than in Trial A (current dispatch orders), reflecting reduced mental workload. Based on a two-tailed α = 0.05, β = 0.2 (power of 80%) and an expected effect size of r = 0.5, a sample size of 32 was estimated for the Wilcoxon signed-rank test. To allow for possible data exclusion, we recruited 35 participants. No interim analyses or early stopping criteria were applied during the study.

### Statistical Analysis

This study aimed to compare participants’ physiological responses and subjective workload with and without the use of the proposed decision-support interface. Paired analyses were conducted to compare HRV and NASA-TLX scores between the two trial conditions. For normally distributed data, paired *t*-tests were used; otherwise, the Wilcoxon signed-rank test was applied. The Shapiro–Wilk test was used to assess the normality of score distributions and their differences. To examine associations between physiological and subjective workload measures, we used the Pearson correlation for normally distributed data and Spearman correlation for non-normal data. The System Usability Scale score, collected only after the decision-support condition, was summarized descriptively.

We conducted subgroup analyses to explore the influence of sex, education level, age, and years of service on usability and workload outcomes. These categories were derived based on the distribution of the collected sample and were not prespecified. The analyses were exploratory and aimed to inform future implementation strategies. All statistical analyses were performed using Minilab (version 3.13; Minitab, LLC., State College, PA) and Python (version 18.1; Python Software Foundation, Wilmington, DE). Results were reported as mean (standard deviation) or median (Q1–Q3), depending on data distribution. A two-tailed *P* value of < .05 was considered statistically significant.

## RESULTS

We initially identified 46 potential participants, of whom 35 EMT-intermediate-certified personnel completed informed consent and participated. Participants were randomized into two groups: Trial A first (current dispatch orders, *n* = 18) or Trial B first (the proposed decision-support interface, *n* = 17). All participants completed both trials, and no dropouts or adverse events occurred (Figure).

Participant demographics included 31 males and four females. A total of 21 participants held a university degree (4-year independent college) and 14 held a college degree (2-to-5-year community college). The mean age was 27.5 (5.34) years. The average years of service was 1.33 (0.81) years (Table 1).

Paired analyses revealed significant differences in multiple HRV indicators between Trial A and Trial B, indicating reduced mental workload during proposed interface use. Specifically, the root mean square of successive differences increased significantly from 28.11 (13.01) milliseconds in Trial A to 32.40 (13.57) milliseconds in Trial B (mean difference 4.28; 95% CI, 1.57–6.99; *Z* = 3.26, *P* < .01), representing the primary outcome. The mean RR increased (784.19 [89.46] vs 808.94 [87.34]; mean difference 24.75; 95% CI, 11.71–37.79; *Z* = 3.21, *P* < .01), while mHR decreased (77.46 [8.61] vs 73.79 [10.62]; mean difference −3.67; 95% CI, −6.43 to −0.90; *Z* = −3.38, *P* < .01), and HF increased (370.10 [291.66] to 482.57 [433.87]; mean difference 112.46; 95% CI, 15.35–209.57; *Z* = 2.43, *P* = .02) all showed significant changes in the expected direction. In contrast, pNN50 (11.39 [11.79] vs 13.45 [13.95]; mean difference 2.06; 95% CI, −0.48 to 4.61; *Z* = 1.48, *P* = .14), LF (686.84 [577.52] vs 850.76 [733.76]; mean difference 163.92; 95% CI, 8.97–318.87; *Z* = 1.76, *P* = .08), and LF/HF ratio (2.33 [1.35] vs 2.16 [1.25]; mean difference −0.17; 95% CI, −0.59 to 0.25; *Z* = −1.36, *P* = .17) did not reach significance (Table 2). Low-frequency power values increased slightly, but the trend was inconsistent with other HRV parameters.

The total NASA-TLX score, measured on a 0–100 scale, did not significantly deviate from normality, as assessed by the Shapiro–Wilk test (*P* > .05). A paired-sample *t*-test revealed a significant reduction in subjective workload when using the decision-support interface. The mean NASA-TLX score decreased from 62.54 (14.61) in Trial A to 35.45 (16.97) in Trial B, representing an average reduction of 26.04 points (95% CI, −34.23 to −20.02; standard deviation (SD) = 22.07), which was statistically significant (*t* = −6.98, *P* < .01).

Subgroup analyses were conducted to explore the potential influence of participant characteristics—sex, education level, age, and years of service—on usability and workload outcomes. These categories were derived from the actual distribution of the sample and not prespecified prior to data collection. No significant sex differences were found in HRV or NASA-TLX scores. However, this finding should be interpreted cautiously because the small number of female participants may have limited the statistical power to detect sex-related differences. Participants with a university degree showed a significantly larger decrease in LF/HF ratio compared to those with a college degree (*P* = .05). Younger participants (aged 20–26) exhibited significantly greater reductions in mHR (*P* = .03), LF (*P* = .02), and LF/HF (*P* = .03), indicating stronger autonomic responses (Supplementary Table 1). Differences by years of service were non-significant, though small-to-moderate effect sizes were observed in the root mean square of successive differences and LF.

For NASA-TLX, no subgroup differences reached statistical significance, suggesting consistent workload reduction across demographic groups (Supplementary Table 2). Two-way repeated measures analysis of variance revealed no significant trial order effects for any HRV or NASA-TLX outcome (all *P* > .05) (Supplementary Table 3). Correlation analyses showed that changes in the root mean square of successive differences, mean RR, mHR, and LF were significantly associated with reductions in NASA-TLX scores (all *P* < .05), supporting the validity of physiological indicators (Supplementary Table 4).

System usability was assessed using the System Usability Scale scoring range from 0–100, with higher scores indicating better perceived usability. In this study the scale was administered only after completion of the decision-support condition. The mean score was 78.07 (16.76), and the median was 80 (interquartile range [IQR], 72.50–90.00), indicating generally positive user perceptions. Based on the System Usability Score grading scale, 9 participants rated the system in the A range (90–100), 10 rated it in the B or C range (70–89), 1 rated it in the D range (60–69), and 5 gave it an F rating (<60), suggesting limited usability concerns (Table 3).

Subgroup analyses examined potential differences in System Usability Scale scores across demographic subgroups. Shapiro–Wilk tests indicated non-normal distributions for sex and education, leading to Mann–Whitney U tests, while age and years of service were analyzed using independent *t*-tests. The System Usability Scale scores did not significantly differ by sex, age, or education level. However, participants with 1.0–1.5 years of service reported significantly higher scores than those with 1.5–3.0 years (*t* = 2.43, *P* = .02), suggesting that less experienced EMTs perceived the system as more usable (Supplementary Table 5).

## DISCUSSION

In this study, use of the decision-support interface for hospital selection was associated with significantly reduced mental workload among EMTs, as reflected by improvements in both physiological and subjective measures. The primary HRV outcome, root mean square of successive differences, increased significantly during interface use, suggesting reduced physiologic strain and improved beat-to-beat variability. Increased mean RR and decreased mHR were also observed, consistent with reduced physiologic demand during decision-making. These physiologic findings were consistent with subjective workload results, as NASA-TLX scores demonstrated a substantial reduction in perceived workload during interface use.

In the frequency domain, both LF and HF power increased while the LF/HF ratio decreased. However, LF interpretation remains complex because LF is influenced by multiple physiologic mechanisms and does not exclusively reflect sympathetic activity.^21^ Therefore, these findings should be interpreted cautiously as supportive evidence of altered autonomic regulation rather than definitive indicators of sympathetic or parasympathetic dominance. Given the simulation-based design and absence of controlled respiratory measurements, causal physiologic interpretations cannot be definitively established.

Overall, the consistency between HRV and NASA-TLX findings suggests that the decision-support interface may help reduce cognitive burden during prehospital stroke triage by simplifying hospital selection and presenting treatment-related information in a structured format. In addition, significant correlations between changes in HRV parameters and NASA-TLX scores further support the convergent validity of these physiologic and subjective workload measures.

These results support previous research showing that real-time decision support can help mitigate mental burden and reduce errors in high-stakes prehospital environments.^22^ In our simulation-based study, use of the decision-support interface was associated with reduced mental workload and concurrent changes in HRV measures among EMTs during stroke triage.

Usability scores were generally positive (mean System Usability Scale score = 78.10), indicating that the interface design met the practical needs of EMTs in hospital selection scenarios. This is consistent with prior findings that well-designed interfaces—those that align with real-world workflows—are crucial for adoption and effectiveness in emergency settings.^23^ Subgroup analysis further revealed that EMTs with shorter field experience (1.0–1.5 years) reported significantly higher scores than those with 1.5–3.0 years of service (*t* = 2.43, *P* = .02), suggesting greater openness to technological tools among early-career personnel. This finding is consistent with broader patterns in healthcare technology adoption, where tenure and familiarity with legacy systems can dampen receptiveness to innovation.^24^

## LIMITATIONS

There are some limitations to this study. First, the decision-support model was built on retrospective data and may not fully reflect complex on-scene decisions. Second, participants were drawn from only one EMS in an urban area with high hospital density, which may limit generalizability to rural regions. Third, the study focused on EMTs with fewer than three years of service to better evaluate mental workload during stroke triage among less experienced personnel; therefore, the findings may not be fully generalizable to more experienced EMTs. In addition, the relatively modest sample size may limit generalizability; however, the randomized crossover design enabled within-subject comparisons that may reduce interindividual variability. Finally, although both objective and subjective indicators were used, potential interindividual variability and context-specific factors (eg, team dynamics) were not fully captured.

Despite these limitations, this study provides preliminary evidence that decision-support interfaces may help reduce mental burden during prehospital stroke triage. Future research should evaluate human–system interactions, integrate these tools into operational dispatch systems, and assess their performance across diverse clinical environments, including rural settings.

## CONCLUSION

This study demonstrated that integrating a web-based decision-support interface effectively reduces mental workload and psychological stress among EMTs during prehospital stroke hospital selection. Both physiological and subjective measures confirmed decreased mental workload and increased parasympathetic activity, indicating reduced stress and improved autonomic balance. The positive user experience further supports the feasibility of implementing such tools in real-world emergency settings. Future studies should validate system effectiveness under operational EMS conditions and explore integration pathways that support adaptive modeling, human–artificial intelligence interaction, and deployment in diverse healthcare environments.

## Supplementary Information











## Figures and Tables

**Figure:**
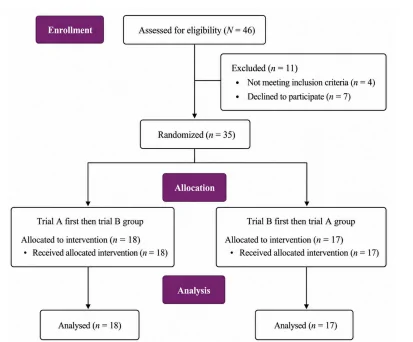
Flow diagram of study participants in a randomized crossover study of a decision-support interface for prehospital stroke triage among emergency medical technicians. Trial A: current dispatch orders; Trial B: the proposed decision-support interface.

**Table 1:** Characteristics of participants in a randomized crossover study of a decision-support interface for prehospital stroke triage among emergency medical technicians.

	*n*	Percentage
Sex		
Male	31	89 %
Female	4	11 %
Education level		
University	21	60 %
College	14	40 %
Age		
20 – 25	14	40 %
26 – 30	12	34 %
31 – 35	5	14 %
36 – 39	4	11 %
Years of Service		
<1	10	29 %
1 – 2	20	57 %
2 – 3	5	14 %
Total	35	100 %

**Table 2:** Differences in heart rate variability indicators between Trial A and Trial B in a randomized crossover study evaluating a decision-support interface for prehospital stroke triage among emergency medical technicians.

	Trial A Mean (SD)	Trial B Mean (SD)	*Z* value	*P* value	Expected Trend	Observed Trend
RMSSD	28.11 (13.01)	32.40 (13.57)	3.26	< .01	↑	✓
mRR	784.19 (89.46)	808.94 (87.34)	3.21	< .01	↓	✓
mHR	77.46 (8.61)	73.79 (10.62)	−3.38	< .01	↑	✓
pNN50	11.39 (11.79)	13.45 (13.95)	1.48	.14	↑	✓
LF	686.84 (577.52)	850.76 (733.76)	1.76	.08	↓	x
HF	370.10 (291.66)	482.57 (433.87)	2.43	.02	↑	✓
LF/HF	2.33 (1.35)	2.16 (1.25)	−1.36	.17	↓	✓

✓ indicates consistency with the expected trend under reduced mental workload; ✗ indicates inconsistency.

*HF*, high-frequency power; *LF*, low-frequency power; *mHR*, mean heart rate; *mRR*, mean R–R interval; *pNN50*, percentage of successive R–R intervals differing by more than 50 milliseconds; *RMSSD*, root mean square of successive differences; *SD*, standard deviation.

**Table 3:** System Usability Scale score range and statistics results in a randomized crossover study evaluating a decision-support interface for prehospital stroke triage among emergency medical technicians.

Grade Scale	Score Range	*n*	Percentage	Mean (SD)	Median (IQR)
A	90 – 100	9	25.71%	96.11 (4.17)	95 (92.5 – 100)
B	80 – 89	10	28.57%	83.25 (3.13)	83.75 (80 – 85.63)
C	70 – 79	10	28.57%	74.75 (1.85)	75 (72.5 – 75.63)
D	60 – 69	1	2.86%	60.00	60 (* – *)
F	0 – 59	5	14.29%	45.50 (9.42)	47.5 (37.5 – 52.5)
Total	0 – 100	35	100%	78.07 (16.76)	80 (72.50 – 90.00)

System Usability Scale scores were interpreted according to established guidelines: <50 = not acceptable (F), 50–69 = marginal (D–C), 70–79 = good (C), 80–89 = excellent (B), and ≥90 = best imaginable (A).

*IQR*, interquartile range; *SD*, standard deviation.

## References

[1] World Health Organization (2020). The Top 10 Causes of Death.

[2] Tsao CW, Aday AW, Almarzooq ZI (2022). Heart disease and stroke statistics—2022 update: A report from the American Heart Association. Circulation.

[3] Campbell BC, Mitchell PJ, Kleinig TJ (2015). Endovascular therapy for ischemic stroke with perfusion-imaging selection. N Engl J Med.

[4] Sheth SA, Jahan R, Gralla J (2015). Time to endovascular reperfusion and degree of disability in acute stroke. Ann Neurol.

[5] Lees KR, Bluhmki E, von Kummer R (2010). Time to treatment with intravenous alteplase and outcome in stroke: An updated pooled analysis of ECASS, ATLANTIS, NINDS, and EPITHET trials. Lancet.

[6] Wang CH, Chang YC, Yang Y (2022). Prehospital-stroke-scale parameterized hospital selection protocol for suspected stroke patients considering door-to-treatment durations. J Am Heart Assoc.

[7] Katsanos AH, Poppe A, Swartz RH (2024). Interhospital transfer for endovascular stroke treatment in Canada: results from the OPTIMISE registry. Stroke.

[8] Jauch EC, Schwamm LH, Panagos PD (2021). Recommendations for regional stroke destination plans in rural, suburban, and urban communities from the Prehospital Stroke System of Care Consensus conference: a consensus statement from the American Academy of Neurology, American Heart Association/American Stroke Association, American Society of Neuroradiology, National Association of EMS Physicians, National Association of State EMS Officials, Society of NeuroInterventional Surgery, and Society of Vascular and Interventional Neurology; endorsed by the Neurocritical Care Society. Stroke.

[9] Hsieh MJ, Lin CJ, Lin YH (2024). An optimization model for reducing thrombectomy center rotations while maintaining medical accessibility. J Formos Med Assoc.

[10] Cheng YC, Yang Y, Wang CH (2025). Hospital selection for suspected stroke: Risk-averse approach considering the minimal risk of exceeding the therapeutic time window. J Neurointerv Surg.

[11] Hopewell S, Chan AW, Collins GS (2025). CONSORT 2025 explanation and elaboration: updated guideline for reporting randomised trials. BMJ.

[12] Lin KW, Chen YJ, Hou SW (2023). Effect of using G-FAST to recognize emergent large vessel occlusion: a city-wide community experience. J Formos Med Assoc.

[13] Hsieh MJ, Tang SC, Chiang WC (2016). Effect of prehospital notification on acute stroke care: a multicenter study. Scand J Trauma Resusc Emerg Med.

[14] Delaney JP (2002). The Effects of Stress and Relaxation on Heart Rate Variability in Health and Disease. PhD thesis.

[15] Rajendra Acharya U, Paul Joseph K, Kannathal N (2006). Heart rate variability: a review. Med Biol Eng Comput.

[16] Hart SG, Staveland LE, Hancock PA, Meshkati N (1988). Development of NASA-TLX (task load index): results of empirical and theoretical research. Human mental workload.

[17] Bangor A, Kortum PT, Miller JT (2008). An empirical evaluation of the System Usability Scale. Int J Hum Comput Interact.

[18] Schaffarczyk M, Rogers B, Reer R (2022). Validity of the Polar H10 sensor for heart rate variability analysis during resting state and incremental exercise in recreational men and women. Sensors (Basel).

[19] Yao Y, Lian Z, Liu W (2009). Heart rate variation and electroencephalograph—the potential physiological factors for thermal comfort study. Indoor Air.

[20] Brooke J, Jordan PW, Thomas B, Weerdmeester BA, McClelland IL (1996). SUS: a “quick and dirty” usability scale. Usability evaluation in industry.

[21] Goldstein DS, Bentho O, Park MY (2011). Low-frequency power of heart rate variability is not a measure of cardiac sympathetic tone but may be a measure of modulation of cardiac autonomic outflows by baroreflexes. Exp Physiol.

[22] Gaynor M, Seltzer M, Moulton S, Sunderam VS, van Albada GD, Sloot PMA, Dongarra JJ (2005). A dynamic, data-driven, decision support system for emergency medical services. Computational science – ICCS 2005.

[23] Kapalo K, Bonnell J, Laviola J (2020). Outside the box: contextualizing user experience challenges in emergency medical technician (EMT) and paramedic workflows. Proc Hum Factors Ergon Soc Annu Meet.

[24] Eckhaus E, Iholkina V, Shkolnik E (2022). The impact of healthcare executive seniority on implementation of innovative methods of diagnosis and prevention. Health Policy.

