# Supplementary material for: Impact of a Decision-Support Interface on Mental Workload and Stress in Prehospital Stroke Triage: A Randomized Crossover Trial
Source: West J Emerg Med. 2026 Sep 5;27(5):1428–35. doi: 10.5811/westjem.61934 (PMC13660630; doi:10.5811/westjem.61934)
Supplement: Supplementary file 1 [file wjem-27-1428-s001.docx]

Supplementary Table 1. Subgroup analysis of differences of heart rate variability indicators between two trials by sex, education level, age, and years of service, in a randomized crossover study evaluating a decision-support interface for prehospital stroke triage among emergency medical technicians.

|  | Group | n | Trial A Mean (SD) | Trial B Mean (SD) | *Test Statistic* | *P*-value |
| --- | --- | --- | --- | --- | --- | --- |
| *RMSSD* |  |  |  |  |  |  |
| Gender | Male | 31 | 26.82 (11.60) | 31.50 (12.46) | Z = .67 | .53 |
|  | Female | 4 | 38.11 (20.54) | 39.39 (21.47) |  |  |
| Education Level | University | 21 | 26.31 (11.69) | 31.35 (12.58) | Z = .30 | .78 |
|  | College | 14 | 30.82 (14.81) | 33.97 (15.28) |  |  |
| Age | 20 – 26 | 17 | 32.21 (12.53) | 36.34 (13.55) | Z = .40 | .70 |
|  | 27 – 39 | 18 | 24.24 (12.57) | 28.68 (12.85) |  |  |
| Years of service | 0 – 1.5 | 18 | 29.27 (14.06) | 35.25 (13.46) | t = 1.33 | .19 |
|  | 1.5 – 3.0 | 17 | 26.89 (12.11) | 29.37 (13.42) |  |  |
| *mRR* |  |  |  |  |  |  |
| Gender | Male | 31 | 780.85 (81.94) | 809.12 (79.89) | Z = 1.50 | .14 |
|  | Female | 4 | 810.07 (150.22) | 807.56 (150.41) |  |  |
| Education Level | University | 21 | 781.87 (83.44) | 805.93 (78.99) | t = -0.13 | .90 |
|  | College | 14 | 787.67 (101.00) | 813.45 (101.56) |  |  |
| Age | 20 – 26 | 17 | 773.97 (92.81) | 809.79 (87.03) | t = 1.72 | .09 |
|  | 27 – 39 | 18 | 793.84 (87.73) | 808.14 (90.14) |  |  |
| Years of service | 0 – 1.5 | 18 | 795.65 (84.03) | 830.70 (76.57) | Z = 1.52 | .13 |
|  | 1.5 – 3.0 | 17 | 772.05 (95.92) | 785.90 (94.25) |  |  |
| *mHR* |  |  |  |  |  |  |
| Gender | Male | 31 | 77.66 (8.14) | 73.48 (10.41) | Z = -1.50 | .14 |
|  | Female | 4 | 75.89 (13.17) | 76.20 (13.67) |  |  |
| Education Level | University | 21 | 77.57 (8.27) | 75.14 (7.43) | Z = 0.54 | .60 |
|  | College | 14 | 77.28 (9.41) | 71.76 (14.24) |  |  |
| Age | 20 – 26 | 17 | 78.51 (8.83) | 72.34 (12.43) | Z = -2.15 | .03 |
|  | 27 – 39 | 18 | 76.47 (8.53) | 75.16 (8.72) |  |  |
| Years of service | 0 – 1.5 | 18 | 76.13 (7.31) | 70.40 (11.03) | Z = -1.85 | .07 |
|  | 1.5 – 3.0 | 17 | 78.86 (9.84) | 77.37 (9.16) |  |  |
| *pNN50* |  |  |  |  |  |  |
| Gender | Male | 31 | 10.10 (9.15) | 12.24 (12.01) | Z = -.31 | .78 |
|  | Female | 4 | 21.43 (24.25) | 22.87 (25.12) |  |  |
| Education Level | University | 21 | 9.17 (9.16) | 11.63 (12.18) | Z = .34 | .75 |
|  | College | 14 | 14.73 (14.66) | 16.19 (16.35) |  |  |
| Age | 20 – 26 | 17 | 12.37 (13.45) | 16.75 (15.43) | Z = 1.91 | .06 |
|  | 27 – 39 | 18 | 10.47 (10.30) | 10.34 (12.01) |  |  |
| Years of service | 0 – 1.5 | 18 | 14.17 (13.47) | 16.22 (15.25) | Z = .23 | .83 |
|  | 1.5 – 3.0 | 17 | 8.45 (9.21) | 10.52 (12.20) |  |  |
| *LF* |  |  |  |  |  |  |
| Gender | Male | 31 | 632.82 (521.26) | 816.62 (715.20) | Z = .73 | .50 |
|  | Female | 4 | 1105.55 (895.33) | 1115.38 (938.78) |  |  |
| Education Level | University | 21 | 704.19 (561.89) | 852.45 (835.33) | Z = -.81 | .32 |
|  | College | 14 | 660.83 (621.28) | 848.23 (578.46) |  |  |
| Age | 20 – 26 | 17 | 750.08 (601.31) | 1089.23 (808.21) | t = 2.32 | .03 |
|  | 27 – 39 | 18 | 627.12 (565.20) | 625.55 (592.83) |  |  |
| Years of service | 0 – 1.5 | 18 | 744.92 (580.88) | 1043.35 (790.36) | t = 2.10 | .04 |
|  | 1.5 – 3.0 | 17 | 625.36 (585.60) | 646.85 (628.08) |  |  |
| *HF* |  |  |  |  |  |  |
| Gender | Male | 31 | 318.93 (221.44) | 439.36 (397.56) | t = 1.25 | .27 |
|  | Female | 4 | 766.66 (486.79) | 817.42 (619.75) |  |  |
| Education Level | University | 21 | 304.63 (213.18) | 469.09 (448.08) | t = 1.44 | .16 |
|  | College | 14 | 468.32 (367.76) | 502.79 (427.44) |  |  |
| Age | 20 – 26 | 17 | 452.53 (329.03) | 585.28 (496.59) | t = -.69 | .50 |
|  | 27 – 39 | 18 | 292.25 (234.57) | 385.56 (351.95) |  |  |
| Years of service | 0 – 1.5 | 18 | 438.25 (327.74) | 575.47 (481.70) | t = .51 | .62 |
|  | 1.5 – 3.0 | 17 | 297.95 (236.39) | 384.20 (365.36) |  |  |
| *LF/HF* |  |  |  |  |  |  |
| Gender | Male | 31 | 2.39 (1.38) | 2.21 (1.27) | t = -.14 | .89 |
|  | Female | 4 | 1.89 (1.13) | 1.80 (1.13) |  |  |
| Education Level | University | 21 | 2.60 (1.32) | 2.16 (1.34) | Z = -2.02 | .05 |
|  | College | 14 | 1.93 (1.33) | 2.17 (1.14) |  |  |
| Age | 20 – 26 | 17 | 2.05 (1.51) | 2.35 (1.33) | t = 2.32 | .03 |
|  | 27 – 39 | 18 | 2.59 (1.15) | 1.98 (1.17) |  |  |
| Years of service | 0 – 1.5 | 18 | 2.29 (1.63) | 2.33 (1.37) | t = 1.05 | .30 |
|  | 1.5 – 3.0 | 17 | 2.38 (1.01) | 1.99 (1.12) |  |  |

Note: Mann-Whitney U test or independent-samples t-test was applied depending on data distribution. *HF*, high-frequency power; *LF*, low-frequency power; *LF/HF*, low-frequency to high-frequency power ratio; *mHR*, mean heart rate; *mRR*, mean R–R interval; *pNN50*, percentage of successive R–R intervals differing by more than 50 ms; *RMSSD*, root mean square of successive differences; *SD*, standard deviation.
