# Supplementary material for: Impact of a Decision-Support Interface on Mental Workload and Stress in Prehospital Stroke Triage: A Randomized Crossover Trial
Source: West J Emerg Med. 2026 Sep 5;27(5):1428–35. doi: 10.5811/westjem.61934 (PMC13660630; doi:10.5811/westjem.61934)
Supplement: Supplementary file 2 [file wjem-27-1428-s002.docx]

Supplementary Table 2. Subgroup analysis of NASA-task load index score changes between Trial A and Trial B by sex, education level, age, and years of service, in a randomized crossover study evaluating a decision-support interface for prehospital stroke triage among emergency medical technicians.

|  | Group | n | Trial A Mean (SD) | Trial B Mean (SD) | *Z-value* | *P-value* |
| --- | --- | --- | --- | --- | --- | --- |
| Gender | Male | 31 | 63.77 (14.39) | 35.16 (17.04) | -1.50 | .14 |
|  | Female | 4 | 63.38 (16.89) | 47.75 (18.55) |  |  |
| Education Level | University | 21 | 63.19 (14.46) | 35.98 (17.58) | -.02 | 1.00 |
|  | College | 14 | 64.54 (14.86) | 37.54 (17.77) |  |  |
| Age | 20 – 26 | 17 | 67.74 (13.22) | 39.59 (17.01) | -.22 | .84 |
|  | 27 – 39 | 18 | 59.94 (14.83) | 33.78 (17.80) |  |  |
| Years of Service | 0 – 1.5 | 18 | 64.86 (14.86) | 34.42 (17.30) | -.74 | .47 |
|  | 1.5 – 3 | 17 | 62.53 (14.29) | 38.91 (17.76) |  |  |

Note: *SD*, standard deviation.
