# Supplementary material for: Impact of a Decision-Support Interface on Mental Workload and Stress in Prehospital Stroke Triage: A Randomized Crossover Trial
Source: West J Emerg Med. 2026 Sep 5;27(5):1428–35. doi: 10.5811/westjem.61934 (PMC13660630; doi:10.5811/westjem.61934)
Supplement: Supplementary file 3 [file wjem-27-1428-s003.docx]

Supplementary Table 3. Two-Way repeated measures ANOVA results on heart rate variability and NASA-task load index for order effects and trial order interaction, in a randomized crossover study evaluating a decision-support interface for prehospital stroke triage among emergency medical technicians.

|  | *F-value* | *P-value* |
| --- | --- | --- |
| mRR | .38 | .54 |
| mHR | .97 | .33 |
| RMSSD | .09 | .77 |
| pNN50 | .50 | .48 |
| LF | .56 | .46 |
| HF | .17 | .68 |
| LF/HF | .22 | .64 |
| NASA-TLX | .05 | .82 |

Note: *HF*, high-frequency power; *LF*, low-frequency power; *LF/HF*, low-frequency to high-frequency power ratio; *mHR*, mean heart rate; *mRR*, mean R–R interval; *pNN50*, percentage of successive R–R intervals differing by more than 50 ms; *RMSSD*, root mean square of successive differences; *SD*, standard deviation.
