# Supplementary material for: Impact of a Decision-Support Interface on Mental Workload and Stress in Prehospital Stroke Triage: A Randomized Crossover Trial
Source: West J Emerg Med. 2026 Sep 5;27(5):1428–35. doi: 10.5811/westjem.61934 (PMC13660630; doi:10.5811/westjem.61934)
Supplement: Supplementary file 4 [file wjem-27-1428-s004.docx]

Supplementary Table 4. Correlation of heart rate variability and NASA-task load index differences between Trial A and Trial B, in a randomized crossover study evaluating a decision-support interface for prehospital stroke triage among emergency medical technicians.

|  | RMSSD | mRR | mHR | pNN50 | LF | HF | LF/HF |
| --- | --- | --- | --- | --- | --- | --- | --- |
| *Spearman rho* | -.54 | -.35 | .41 | -.28 | -.39 | -.28 | -.17 |
| *P-value* | <.01 | .04 | .01 | .10 | .02 | .10 | .34 |

Note: *HF*, high-frequency power; *LF*, low-frequency power; *LF/HF*, low-frequency to high-frequency power ratio; *mHR*, mean heart rate; *mRR*, mean R–R interval; *pNN50,* percentage of successive R–R intervals differing by more than 50 ms; *RMSSD*, root mean square of successive differences; *SD*, standard deviation.
