# Supplementary material for: Impact of a Decision-Support Interface on Mental Workload and Stress in Prehospital Stroke Triage: A Randomized Crossover Trial
Source: West J Emerg Med. 2026 Sep 5;27(5):1428–35. doi: 10.5811/westjem.61934 (PMC13660630; doi:10.5811/westjem.61934)
Supplement: Supplementary file 5 [file wjem-27-1428-s005.docx]

Supplementary Table 5. Statistical test of System Usability Scale scores by sex, education level, age and years of service, in a randomized crossover study evaluating a decision-support interface for prehospital stroke triage among emergency medical technicians.

|  | Group | n | Mean (SD) | *Test Statistic* | *P-value* |
| --- | --- | --- | --- | --- | --- |
| Sex | Male | 31 | 77.50 (17.36) | *Z* = -.21 | .86 |
|  | Female | 4 | 82.50 (11.90) |  |  |
| Education Level | University | 21 | 78.33 (19.04) | *Z* = .44 | .67 |
|  | College | 14 | 77.68 (13.28) |  |  |
| Age | 20 – 26 | 17 | 80.88 (9.05) | *t* = .98 | .34 |
|  | 27 – 39 | 18 | 75.42 (21.66) |  |  |
| Years of Service | 0 – 1.5 | 18 | 84.44 (8.47) | *t* = 2.43 | .02 |
|  | 1.5 – 3.0 | 17 | 71.32 (20.66) |  |  |

Note: *SD*, standard deviation.
